# Supplementary material for: Spatial Variation in Stream-Water Bacterial Communities Across Sites Differing in Wild Chinese Giant Salamander Detection Frequency
Source: Biology (Basel). 2026 Sep 18;15(18):1647. doi: 10.3390/biology15181647 (PMC13604818; doi:10.3390/biology15181647)
Supplement: Supplementary file 1 [file biology-15-01647-s001.zip › Data Related to “Spatial Variation in Stream-Water Bacterial Communities across Sites Differing in Wild Chinese Giant Salamander Detection Frequency”/Microbial Data/result/08.FunctionPrediction/picrust2/heatmap/sample/heatmap.sample.PFAM.pdf]

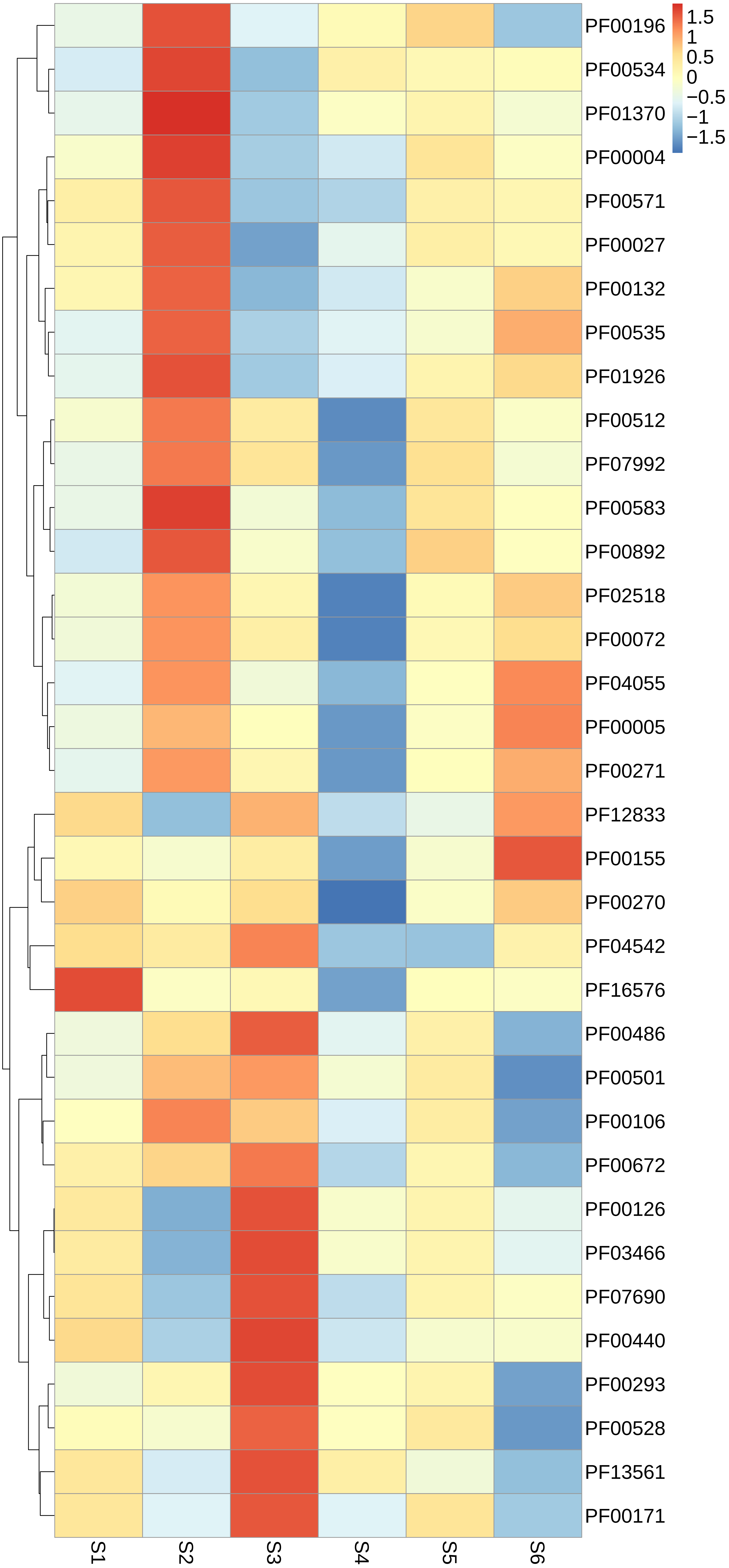

1.5
1
0.5
0
−0.5
−1
−1.5
PF00196
PF00534
PF01370
PF00004
PF00571
PF00027
PF00132
PF00535
PF01926
PF00512
PF07992
PF00583
PF00892
PF02518
PF00072
PF04055
PF00005
PF00271
PF12833
PF00155
PF00270
PF04542
PF16576
PF00486
PF00501
PF00106
PF00672
PF00126
PF03466
PF07690
PF00440
PF00293
PF00528
PF13561
PF00171
S1
S2
S3
S4
S5
S6
